# Enhancing the Fertility Potential: A Case Report on the Management of Oligoasthenozoospermia Using a Microfluidic Device

**DOI:** 10.7759/cureus.61737

**Published:** 2024-06-05

**Authors:** Rohit G Bedwal, Nancy Nair, Charu Pareek, Aakash More, Avanti Kalbande

**Affiliations:** 1 Clinical Embryology, Datta Meghe Institute of Higher Education and Research, Wardha, IND; 2 Anatomy, Datta Meghe Institute of Higher Education and Research, Wardha, IND; 3 Obstetrics and Gynaecology, Shalinitai Meghe Hospital and Research Centre, Nagpur, IND

**Keywords:** infertility, intracytoplasmic sperm injection, microfluidics, asthenozoospermia, oligozoospermia

## Abstract

Low sperm count and motility in oligoasthenozoospermia present significant challenges to conception. This case report involves a couple, a 28-year-old female and a 35-year-old male, experiencing secondary infertility for four years. The male partner's habits of alcohol consumption and smoking were potential infertility factors. Semen analysis revealed a total sperm count of 10 million/mL, with total motility at 30% and progressive motility at 5%. The couple underwent intracytoplasmic sperm injection (ICSI), using advanced sperm separation techniques to isolate motile and morphologically normal sperm. Despite the suboptimal sperm parameters, this approach resulted in successful fertilization and pregnancy. The female partner’s preparation involved a short antagonist treatment, leading to the retrieval of eight oocytes, seven of which were mature. A positive urine pregnancy test and ultrasound confirmed the pregnancy, with β-hCG at 798 mIU/mL. This case highlights the potential of individualized treatments in managing oligoasthenozoospermia, emphasizing their promise in improving assisted reproductive outcomes despite mixed research results.

## Introduction

Infertility has a substantial effect on individuals of reproductive age globally. Statistical projections indicate that a significant portion of the global population, ranging from 48 million couples to 186 million individuals, is affected by infertility [1]. While discernible causative factors are evident in most cases, a notable subset remains categorized as unexplained infertility. Female partners account for a considerable proportion, ranging from 40% to 55%, of infertility instances, whereas male partners contribute to approximately 20% to 40% of cases. One of the reasons for male infertility is a condition called oligoasthenozoospermia [2].

Oligoasthenozoospermia, a pathological condition affecting sperm, presents in accord with two additional disorders: asthenozoospermia, considered by abnormal sperm motility, where over 60% of sperm exhibit immotility or impaired linear motility, and oligozoospermia, denoting decreased sperm count, falling below the threshold of 15 million per milliliter (mL) [3]. A semen examination can identify it and has multiple causes, including underlying medical conditions, lifestyle choices, and heredity. Treatment choices include medication, assisted reproductive procedures, and lifestyle modifications, depending on the severity or underlying causes. It is a common type of male infertility, and with an increase in clinical patients and advancements in the field of assisted reproductive technology [4].

The microfluidic device selects the best sperm for in vitro fertilization (IVF). Ideally, the best sperm would produce better results. It works on the principles of swim-up preparation. Microfluidics are devices that are used in IVF labs to prepare and screen sperm for intracytoplasmic sperm injection (ICSI) insemination. Microfluidics depend on the sperm showing motility by actively swimming through the membrane filter in the chip [5]. This approach eliminates centrifugation, which some reports claim may stress and perhaps damage sperm membranes, in favour of simulating some characteristics of standard conception. To use microfluidics for ICSI, samples must have a minimum of one million progressively motile sperm per milliliter. As a result, samples with lower motile sperm counts or very poor motility will need to be prepared using the traditional approach [6].

## Case presentation

Patient information

In this case, the couple, a 28-year-old female and a 35-year-old male, presented with a complaint of secondary infertility. The patient was a mother of a five-year-old baby boy. They had been trying to conceive a second child for the past four years.

The female patient's previous hormonal reports were within normal limits, and had regular menstruation cycles of ±28 days. She did not have any known medical issues that could lead to infertility. Both male and female partners did not have a history of thyroid, diabetes, or genetic abnormalities. The male partner had habits of alcohol consumption and smoking for the past 12 years, which could be a possible cause of infertility.

Clinical findings

The physical well-being of the couple was average overall. Vital signs for the patient were within normal limits. The male patient exhibited secondary sexual features that were consistent with testosterone production. Examining the external genitalia, it was revealed that the scrotum and testicles were normal. Physical examination revealed no surgical scars, and the scrotum had no palpable lumps. To rule out the reason for the patient's inability to conceive, semen analysis was advised.

Diagnostic assessment

The patient was advised to undergo semen analysis when he enrolled at our center. The sample of semen was collected in the laboratory. There were no anatomical or endocrine anomalies, and overall health was satisfactory. Results revealed that the total sperm count was 1.5 mL, slightly more significant than the reference range of 1.4 mL (with a range of 1.3-1.7 mL). The pH level measured 7.5, falling within the acceptable range of 7.2-7.8. However, liquefaction took 24 minutes, slightly longer than the ideal cut-off of less than 30 minutes. In particular, the total sperm count was 10 mL, below the desired level of greater than 15 mL. Total motility was 30%, notably lower than the reference value of 42%, indicating reduced overall sperm motility. Progressive motility was 5%, significantly lower than the expected 30%, signifying diminished forward motility of sperm. Additionally, the morphology assessment revealed that only 5% of the sperm exhibited normal morphology. The male patient's seminal parameters were obtained, which are shown in Table 1.

**Table 1:** Semen analysis parameter mL: milliliter; mil: million; pH: potential of hydrogen

Parameters	Findings	Reference Levels
Volume	1.5	1.4 (1.3-1.7)
pH	7.5	7.2-7.8
Liquefaction	24 minutes	<30 minutes
Total sperm count	10	>15 mil/mL
Total motility	30%	42%
Progressive motility	5%	30%
Morphology	5	>4

Thus, the patient had been diagnosed with secondary infertility due to oligoasthenozoospermia, which is defined as the presence of immotile spermatozoa higher than the normal range and count less than the reference range. This medical condition can affect the spermatozoa's potential to fertilize an ovum, which leads to difficulties in achieving pregnancy.

A blood test assessed female hormone levels, and the result was normal. Transvaginal ultrasonography (USG) was used to investigate the follicles. The endometrium thickness was within a normal range.

Therapeutic intervention

The couple received thorough information on all of the possible reproductive treatment options, including their procedures and potential drawbacks. Before any further interventions were made, both participants gave their informed consent. A fresh semen sample was collected and prepared using a buffer medium. The medium was incubated in an incubator with 5% CO_2_ until it reached a pH of 7.4 and a temperature of 37°C. To wash the sperm, 4 mL of media was added to 2 mL of the semen sample, and the mixture was centrifuged at 1000 rpm for 10 minutes. After centrifugation, the pellet was resuspended in 0.7-0.8 mL of media after properly discarding the supernatant. The microfluidic device was prepared, including priming the device and ensuring its proper working condition. The collected semen sample was then placed into the device. The microfluidic technology was used to isolate motile and morphologically normal sperm. After the separation technique was complete, purified sperm from the device was retrieved.

**Figure 1:**
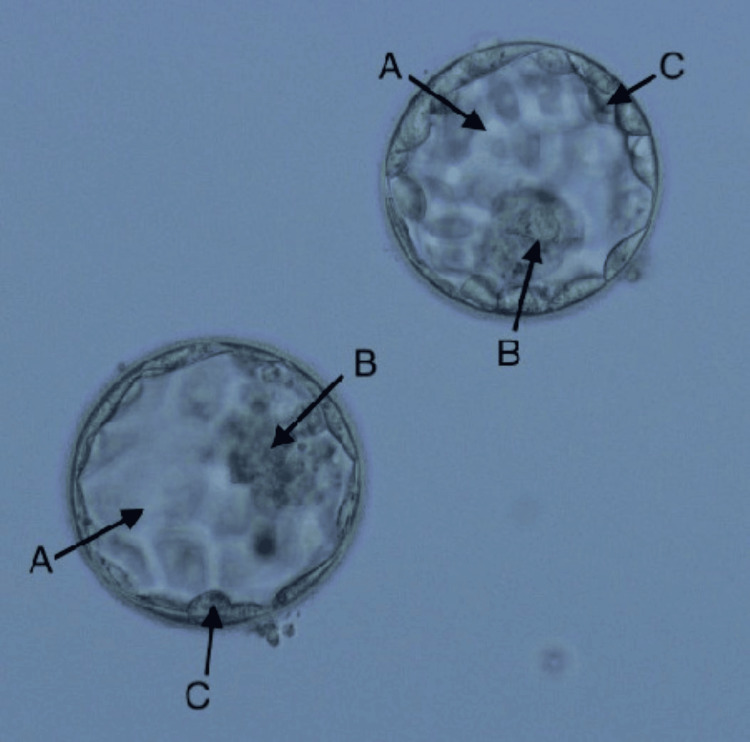
Day 5 blastocyst of grade 4BA and 3AB selected for ET A: 4 and 3 size of the blastocyst (260 µm); B: inner cellular mass; C: trophectoderm; ET: embryo transfer

The female patient's oocyte pickup (OPU) preparation started with a short antagonist treatment. Before the gonadotropin-releasing hormone (GnRH) antagonist was given, the leading follicle was stimulated with follicle-stimulating hormone (FSH)/human menopausal gonadotropin (hMG) to mature to a 14-16 mm diameter. Human chorionic gonadotropin (hCG) was given in combination with 2.5 or 5 mg/d hMG as part of a brief antagonist program to boost ovarian function. To sum up, the GnRH agonist and FSH/hMG were given together as the last triggers in the short protocol. A total of 10,000 IU of hCG was administered 36.5 hours before ovum pickup since the GnRH antagonist induces oocyte maturation. After OPU, a total of eight oocytes were retrieved: seven were metaphase II (MII), and one was germinal vesicle (GV). The purified sperm retrieved from the microfluidic device was used for the ICSI process. Fertilization was checked 17 hours ± 1 hour after the ICSI process; six two pronuclei (2PN) were formed, and by day 5, six blastocysts were formed, which were preserved for further use. The next month, on the day of the scheduled embryo transfer (ET) with an endometrium thickness of 11 mm, day 2 embryos of grade 4BA (B: inner cellular mass grade, A: trophectoderm grade) and 3AB (A: inner cellular mass grade, B: trophectoderm grade) were transferred, as shown in Figure 1.

From day 6, the patient was prescribed progesterone and advised for bed rest.

Follow-up and outcome

Urine pregnancy test (UPT) results were positive after two weeks of ET. A single fetal sac was visible on USG, and the result for β-hCG was 798 mIU/mL.

## Discussion

One of the main reasons for male infertility is oligoasthenozoospermia, which is characterized by low sperm concentration and motility. However, oligoasthenozoospermia has not yet been successfully treated with a medication in modern medicine. In our study also, the male partner was diagnosed with oligoasthenozoospermia, which was addressed through management using microfluidics, resulting in a successful pregnancy outcome.

Robles et al., in their study, found better blastocyst rates and more excellent euploidy rates obtained from a second ICSI cycle utilizing the microfluidic device in patients who had previously undergone an ICSI/preimplantation genetic testing for aneuploidy (PGT-A) cycle employing a density gradient for sperm selection. Even though there were roughly the same amount of mature oocytes and typically fertilized embryos per cycle, these results held. More extensive research is required to verify the results [7].

Vigolo et al., in a study, concluded that in every ejaculate, the microfluidic device selects a specific sperm population; this selection is especially pronounced in frozen-thawed horse semen that has been shown to have low fertility. This crucial finding supports using a microfluid device to quickly and easily select spermatozoa for ICSI. It is advised to utilize the microfluid device for in vitro testing to forecast the fertility of frozen-thawed horse semen because it selects for a specific sperm population and most likely mimics selection systems also present in the female genital tract [8].

There were no discernible differences in fertility treatment outcomes using microfluidics [7,9]. Schiewe et al., in their study, stated that the comparison between traditional sperm wash techniques and the application of the microfluidic device in the general IVF population did not improve the results of embryo development. When ejaculated sperm is feasible, our findings do support the idea that, for male factor patients with elevated DFI (DNA fragmentation index), microfluidic separation of sperm using microfluidics is a more advantageous and economical method than surgically obtaining testicular sperm. However, since our evaluations in this study may be biased by enrolling males with non-obstructive azoospermia, more research is required to clarify the effectiveness of testicular biopsy treatment when inadequate motile and morphologically normal sperm are available in an ejaculate [9].

Zaha et al., in their study, stated that microfluidic devices are currently available as an add-on for IVF and appear to increase the number of high-quality blastocysts and pregnancy rates marginally; however, more research is required to validate this. Furthermore, more investigation is needed to completely comprehend the possible advantages and disadvantages of microfluidic sperm preparation and how it affects the quality of the embryo and the course of pregnancy [10]. Our study emphasizes the difficulty of treating male infertility, especially when it comes to oligoasthenozoospermia, a condition in which sperm motility and count are both reduced. Microfluidics offer a potentially helpful device for treating oligoasthenozoospermia.

## Conclusions

One major obstacle in the treatment of male infertility is oligoasthenozoospermia. Individualized treatments are necessary to address this medical condition, as traditional therapy strategies have minimal success. This case report's use of the microfluidic sperm separation technology offers a potential solution to the challenges caused by oligoasthenozoospermia. The use of microfluidic technology increases the success rates of assisted reproductive procedures by making it easier to isolate motile and morphologically normal sperm. Our study highlights the potential of microfluidics as a valuable tool in the management of male infertility, particularly in cases of oligoasthenozoospermia, despite some inconsistencies in research outcomes. More extensive and prospective trials are necessary to maximize its integration into clinical practice and prove its efficacy. Microfluidics might be a promising development in the realm of assisted reproductive technology for couples who face challenges in conception.
